# Aurophilic Interactions Studied by Quantum Crystallography

**DOI:** 10.1021/acs.inorgchem.1c03333

**Published:** 2022-03-01

**Authors:** Sylwia Pawlȩdzio, Maura Malinska, Florian Kleemiss, Simon Grabowsky, Krzysztof Woźniak

**Affiliations:** † Biological and Chemical Research Centre, Department of Chemistry, University of Warsaw, Żwirki i Wigury 101, 02-089 Warszawa, Poland; ‡ Department of Chemistry, Biochemistry and Pharmaceutical Sciences, University of Bern, Freiestrasse 3, 3012 Bern, Switzerland; § Faculty for Chemistry und Pharmacy, University of Regensburg, Universitätsstrasse 31, 93053 Regensburg, Germany

## Abstract

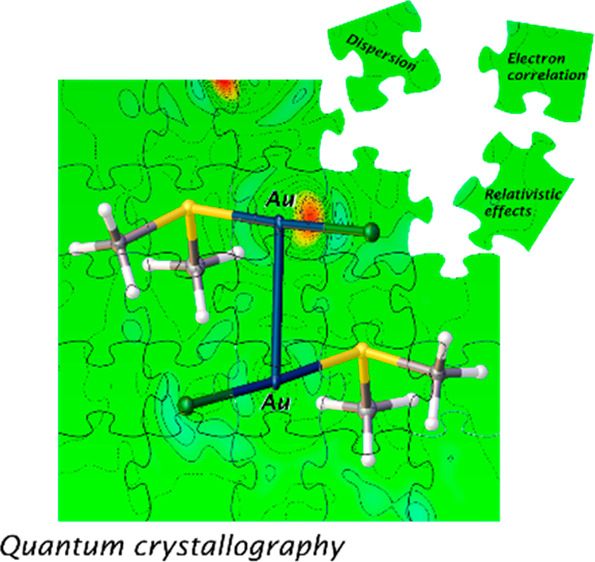

This is the first use of a wave-function-based crystallographic method to characterize aurophilic interactions from X-ray diffraction data. Theoretical calculations previously suggested the importance of electron correlation and dispersion forces, but no influence of relativistic corrections to the Au...Au interaction energy was found. In this study, we confirm the importance of relativistic corrections in the characterization of aurophilic interactions in addition to electron correlation and dispersion.

## Abstract 2

Hirshfeld atom refinement was used to characterize aurophilic interactions from X-ray diffraction data. An intermediate closed-shell type of aurophilic interaction with some features of covalency was identified when both electron correlation and relativistic corrections were applied. Relativistic correction changes the electron density distribution more than electron correlation. Relativistic effects strongly dominate the metal core region also in the direction of the noncovalent interactions and all of the valence and bonding regions with regard to the Au···Au interaction.

Aurophilic interactions^1−3^ refer to weak metallophilic contacts^4−7^ formed between gold atoms with 5d^10^ closed-shell valence electron configuration. They occur as *intra*- or *inter*molecular noncovalent interactions (NCIs) and significantly influence the molecular and crystal structures. They are responsible for unusual aggregations of molecules,^8^ such as, e.g., dimers, trimers, chains, or layers, with short Au...Au distances.^9^ The presence of aurophilic interactions results in the appearance of specific materials properties, including catalytic properties,^10^ electric conductivity,^11^ or luminescence.^12−14^

In all types of metallophilic interactions, the metal–metal distance is shorter than the sum of the van der Waals radii, which is associated with a decrease in the bond energy,^15,16^ comparable to hydrogen or halogen bonds, π–π stacking, and other weak interactions. Generally, metallophilic interactions are formed between metals with low coordination numbers,^17^ typically heavy elements. Therefore, these types of interactions are not only dominated by orbital,^11,18^ electrostatic,^11,18^ and dispersion forces^17^ but also benefit from relativistic effects.^19,20^ At the bottom of the periodic table, relativistic effects are maximal for gold,^21^ and thus the strongest metallophilic interactions are observed for Au···Au contacts.^22−24^

Noncovalent forces play a crucial role in determining both the shape of the molecular structure and the supramolecular architecture of crystals.^25^ A key application of quantum crystallography^26,27^ is to understand the nature of intermolecular NCIs in crystals, going beyond the analysis of geometrical parameters. In that sense, an essential source of information is the electron density, which can be obtained from X-ray diffraction (XRD) experiments and described using quantum mechanical principles and concepts. The quantum crystallographic method utilized here is Hirshfeld atom refinement (HAR; Table 1).^28,29^ HAR is a wave-function-based procedure for modeling diffraction data. Structural parameters, namely atomic positions and their anisotropic displacement parameters (ADPs), are obtained from XRD experiments in an iterative procedure, where cycles of either Hartree–Fock (HF) or density functional theory (DFT) calculations of molecular electron density partitioned into aspherical atoms and least-squares refinement are repeated until convergence. Since the electron density is not refined in HAR but calculated at a high level of theory, the experimentally obtained atomic positions and ADPs from HAR refinement are more accurate and precise than any others.^29,30^ Quantum Crystallography has already been successfully applied to structures containing heavy metals, where the influence of the relativistic effects, electron correlation, and anharmonicity on the electron density distribution in crystals has been confirmed and characterized in detail.^31−34^

**Table 1:** Structure Refinement Details^a^

	IAM	HAR
data/restraints/param	2042/0/48	2042/0/95
GOF on *F*^2^	1.198	0.924
final *R* indices (all data)	*R*_1_ = 2.52%, *wR*_2_ = 6.93%	*R*_1_ = 2.18%, *wR*_2_ = 6.08%
largest diff peak/hole (e/Å^3^)	2.54/–4.17	1.91/–1.65

a Final *R* indices are provided for the IAM and HAR (rks_anh_rel) models refined in *SHEXL* and *NoSpherA2*, respectively.

In practice, theoretical characterization of metallophilic interactions is very difficult because of the complex chemistry of heavy atoms and the lack of accurate experimental references.^35−37^ Thus, the binding energies of aurophilic interactions have been the subject of many theoretical studies at several levels of theory, including DFT, second-order Møller–Plesset (MP2), and coupled cluster singles doubles (triples) [CCSD(T)] methods.^36,38^ It was found that MP2 tends to overestimate the interaction energies with respect to the CCSD(T) reference method.^36^ On the other hand, DFT can only give reasonable dimer energies when a dispersion correction is applied.^35,39,40^

The aim of the present study is to accurately characterize aurophilic interactions present in a crystal structure of chloro(dimethyl sulfide)gold (Figure 1a) by using the HAR method, as implemented in *NoSpherA2*.^41^ The effects of relativity (REL) and electron correlation (ECORR) on the electron density distribution along the Au···Au contact are the main focus of this work. We looked in-depth into the interaction energies, the Quantum Theory of Atoms in Molecules (QTAIM)^42^ parameters at bond critical points (BCPs), and the negative Laplacian profiles of the Cl–Au–S(CH_3_)_2_ dimer (Figure 2) for the theoretical electron densities at the HAR geometries calculated at different levels of theory. The present work is a first attempt at using the wave-function-based method HAR for a description of aurophilic interactions—using its experimental geometry as well as analyzing the wave function used in the refinement.

**Figure 1:**
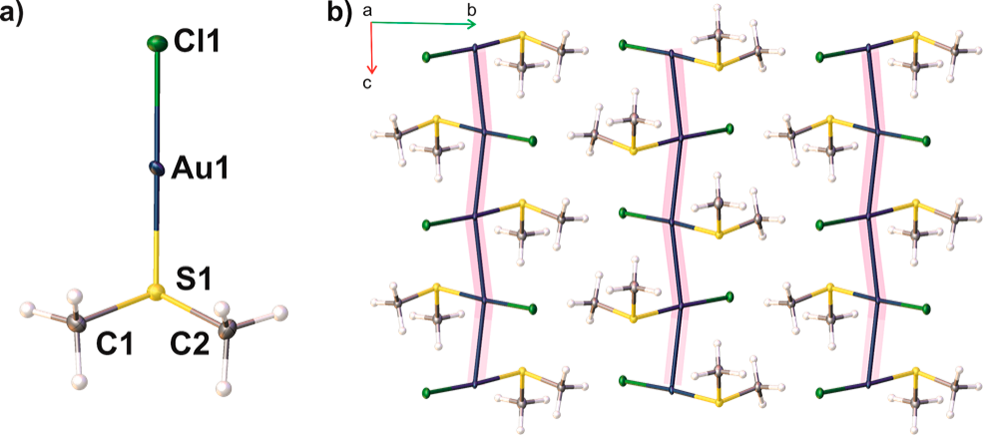
(a) Molecular structure of Cl–Au–S(CH_3_)_2_ after HAR with the labeling scheme. Ellipsoids are drawn with 50% probability. (b) Crystal packing along the [100] crystallographic direction showing aurophilic interactions (highlighted in pink).

**Figure 2:**
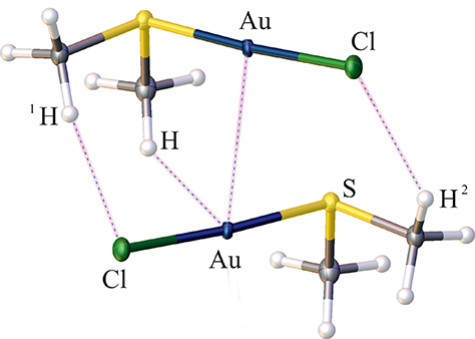
Molecular structure of the investigated Cl–Au–S(CH_3_)_2_ dimer with intermolecular contacts detected within QTAIM analysis. Symmetry codes: 1, *x*, *y*, *z*; 2, *x*, 1.5 – *y*, 0.5 + *z*.

The structure of chloro(dimethyl sulfide)gold^43^ was reinvestigated by collecting the XRD data at the SPring-8 synchrotron station in Japan (beamline BL02B1). The investigated compound crystallized in the monoclinic *P*2_1_/*c* space group with one molecule in the asymmetric unit. All further information about the compound can be found in the Supporting Information (SI). HARs were performed at HF-DKH2/x2c-TZVPPall (rhf_anh_rel, Table 1), B3LYP-DKH2/x2c-TZVPPall (rks_anh_rel, Table 1), and B3LYP/x2c-TZVPPall (rks_anh_nrel). The abbreviation anh refers to the inclusion of anharmonic motion refinement. Empirical dispersion corrections are irrelevant to the electron density underlying HAR; they are only important for calculation of the energies.^44^

The Cl–Au–S atoms are in an exceptional linear geometry, 176.63(2)°, which leaves the coordination sphere of the gold atom open. Molecules form an infinite Au···Au···Au chain in the crystal lattice, along the *c* crystallographic axis (Figure 1b) with a Au···Au···Au valence angle of 167.24(1)°. Adjacent molecules in each Au···Au···Au chain, highlighted in pink (Figure 1), are trans-arranged to each other, with the Cl–Au–S line almost perpendicular to the chain. The Au...Au distance has a typical value of 3.15983(6) Å. The aurophilic interaction energy computed at the B3LYP/x2c-TZVPPall level with empirical dispersion and basis set superposition error corrections at the fixed molecular geometry obtained in HAR (Figure 2) was predicted to be attractive by 67.74 kJ/mol (Table 2).

**Table 2:** Interaction Energies (kJ/mol) for the Cl–Au–S(CH_3_)_2_ Dimer Using the x2c-TZVPPall Basis Set with Counterpoise Correction at Different Levels of Theory

HF-DKH2	2.93
B3LYP-GD3BJ	–67.36
B3LYP-GD3BJ-DKH2	–67.74
B3LYP-DKH2	–12.09

This value seems to be independent of relativistic corrections (B3LYP-GD3BJ-DKH2 vs B3LYP-GD3BJ) but strongly changed when dispersion (B3LYP-DKH2) or electron correlations (HF-DKH2) were neglected. The former reduces the attractive character of the interaction energy, while the latter even makes it repulsive. The studied structure also forms other noncovalent contacts.^45^ More details are given in the SI.

QTAIM was applied to analyze the quantum-mechanical electron density available after HAR. The BCPs and bond paths (Tables S10–S12 and Figure S7) confirm the existence of all covalent bonds as well as C–H···Cl, C–H···Au, and Au···Au intermolecular contacts (Figure 2). Tables 3 and S9 present the QTAIM parameters characterizing the BCPs obtained for the above-mentioned intermolecular contacts at different levels of theory.

**Table 3:** QTAIM Parameters at BCPs for Contacts Present in the Cl–Au–S(CH_3_)_2_ Dimer and DI Computed between Two Atomic Basins

contact	method	ρ(*r*) (e/Å^3^)	∇^2^ρ(*r*) (e/Å^5^)	*V*_r_ (hartree)	*H*_r_/ρ(*r*) (hartree/e)	|*V*_r_|/*G*_r_	DI
Au···Au	rks_anh_rel	0.160	1.398	–0.016	–0.029	1.046	0.25
	rks_anh_nrel	0.125	1.133	–0.011	0.015	0.975	0.16
	rhf_anh_rel	0.140	1.502	–0.015	0.010	0.986	0.18

For all contacts present in Tables 3 and S9, the small values of the electron densities, together with the small and positive values of the corresponding Laplacian, indicate closed-shell interactions. However, the negative sign of the potential energy densities *V*_r_ denotes a stabilizing character of the investigated contacts upon electron sharing. This is confirmed by the delocalization index (DI), which is the average number of electron pairs shared between two atomic basins (Tables 3 and S9). The largest values of the DI among all intermolecular contacts present in the Cl–Au–S(CH_3_)_2_ dimer are found for the aurophilic interactions, indicating their stabilizing character. Stabilization of the Au...Au contact increases when relativistic effects and electron correlation are included (Table 3, model rks_anh_rel), while for Au...H and Cl...H interactions, the influence of these corrections is marginal (Table S9). Within the rks_anh_rel model, a decrease of the total energy density [*H*_r_/ρ(*r*)] to a negative value is observed, and, consequently, some covalent character of the Au···Au contact is detected. Hence, the relativistic effects are a key factor influencing the character of the aurophilic interactions shown in both the DI and *H*_r_/ρ(*r*) parameters. The same trend is observed with regard to the |*V*_r_|/*G*_r_ ratio, which is more sensitive to relativistic effects than the electron correlation correction.^46^ A value > 1 indicates an intermediate strength for an Au...Au interaction.

Electron-density analysis also sheds light on the Au···Au interaction in a more global way. This is shown in Figures 3 and S8 where the distribution of the electron density and its negative Laplacian along the Au···Au contact at different levels of theory are presented. The impact of ECORR and REL is shown in the subplots as subtraction of the rks_anh_rel–rhf_anh_rel and rel_anh_rel–rks_anh_nrel models. Similar to the covalent bonds,^32,34^ the differences between the models are visible. However, the dominant role of ECORR over REL, with regard to the electron density, is only detected in the region of 0.3–0.6 Å (Figure S8).

**Figure 3:**
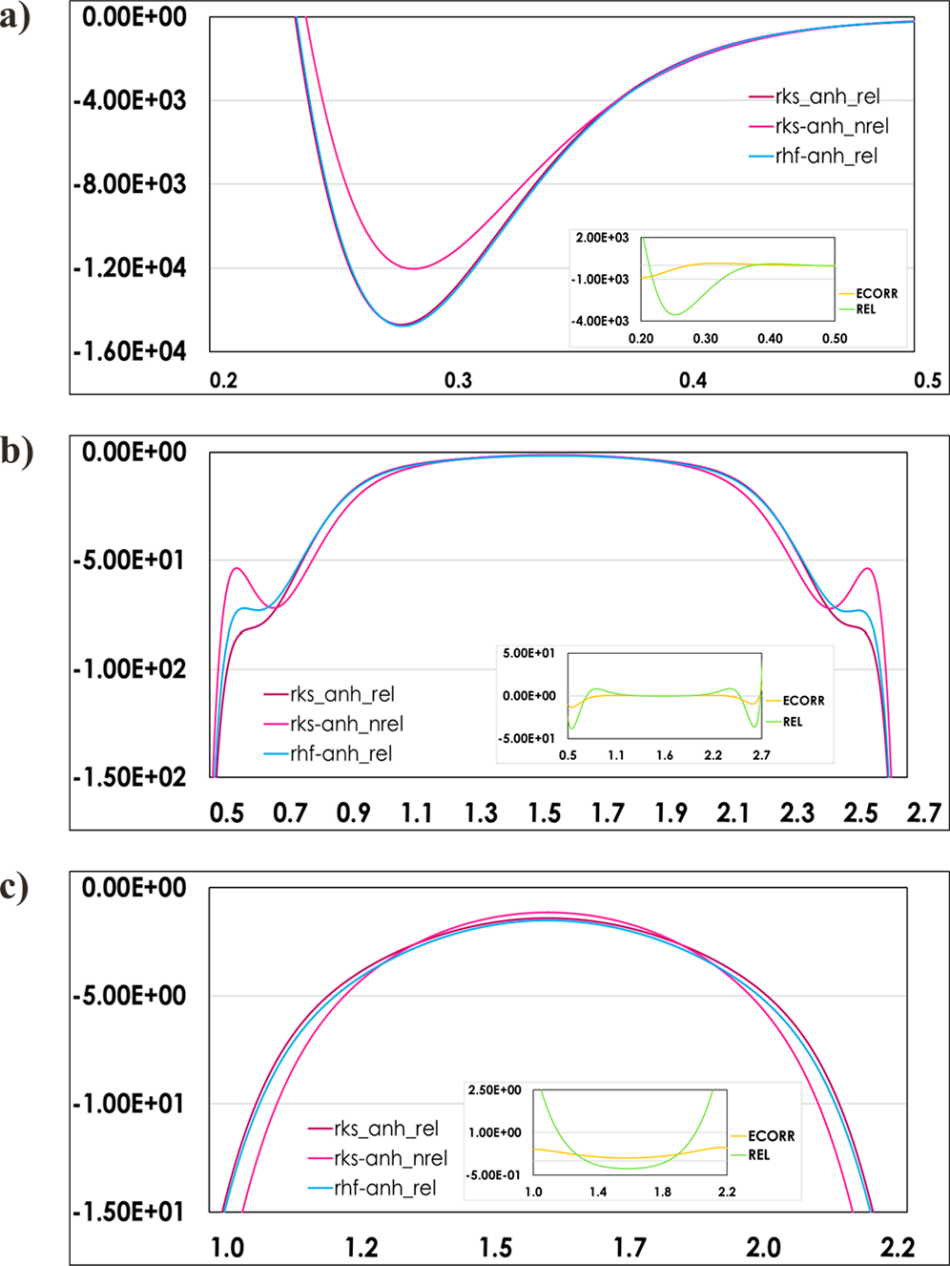
1D plots of negative Laplacian (*y* axis, in e/Å^5^) as a function of the Au···Au contact distance (*x* axis, in Å) for theoretical electron densities used in the HAR models, showing the most interesting changes in their courses in terms of magnifications of different bonding ranges. The subplots show differences of the negative Laplacian resulting from the relativistic (REL) and electron correlation (ECORR) effects obtained as a result of subtraction of the rel_anh_rel–rks_anh_nrel and rks_anh_rel–rhf_anh_rel models, respectively.

The ECORR and REL corrections also influence the shapes of the negative Laplacian profiles. The first difference is visible in the region of local charge depletion (the 0.25–0.35 Å range). Compared to the covalent bonds,^34^ the magnitudes of these minima deviate by ca. 300 and 250 e/Å^5^ for REL and ECORR, respectively, with a shift of the charge depletion minimum due to a relativistic contraction of 0.01 Å. Furthermore, the local charge concentration in the region of 0.5–0.7 Å is the highest for the rks_anh_nrel model (Figure 3). This means that the reduction of the electron density concentration in this region is mainly caused by relativistic effects (similar to the previously reported study for covalent bonds -subplots in Figure 3a): the magnitudes of the local maxima for the Au···Au interaction in this region are half the amounts previously reported for the covalent Au–P and Au–C bonds.^34^ On the other hand, contrary to the covalent bonds, the dependence of the curve of the negative Laplacian on the distance in the bonding region for the Au···Au interaction (1.2 – 2.0 Å) is even more affected by REL (subplots in Figure 3b) than by ECORR.

We have shown that the energy of the dimer is mostly dominated by weak dispersion forces, which are mainly associated with the existence of C–H···Cl, C–H···Au, and C–S···Au contacts and partly enhanced by attractive forces of the Au···Au interaction. Through counterpoise calculations, we have also shown the importance of electron correlation effects on the resulting dimer energy value, which seemed to be independent of the relativistic correction. However, within QTAIM analysis, performed at different levels of theory, we have stressed the importance of both electron correlation and relativistic corrections in the characterization of aurophilic interactions. Within a QTAIM analysis, we have identified an intermediate closed-shell type of aurophilic interaction with some features of covalency but only when REL and ECORR corrections were applied together. Importantly, REL changes the electron density distribution more than ECORR correction. It enhances the stabilization of the Au···Au interactions and their covalent character. The above-mentioned observations correspond well with previously reported studies.^22,33^ Here, we were able to distinguish the significance of dispersion forces and relativistic corrections in terms of dimer energy and electron density properties, respectively.

Dispersion is very important for accurate dimer energy estimation. However, the dimer energy results from all interactions and close contacts present in the dimer, for which other weak contacts such as C–H···Cl, C–H···Au, and C–S···Au prevail over aurophilic interactions. On the other hand, the relativistic correction enhances the covalent character of the Au···Au interaction, which is reflected in the electron density properties.

Relativistic effects strongly dominate the metal core region also in the direction of the NCIs and all of the valence and bonding regions with regard to the metal···metal interaction. Our findings emphasize the computational challenge in the characterization of metal···metal NCIs, in understanding aurophilic and other secondary interactions, and how quantum crystallography can be a very useful tool for deriving reliable bond characterizations for metallophilic interactions.

A geometry optimization for polymeric structures with heavy elements in periodic conditions, with an all-electron basis set, is still a challenge for theoretical chemistry, and thus the geometry obtained by an XRD experiment is very important. This seems to be a great solution in this case because we are able to combine both experimental data (which characterized the geometry in periodic conditions) and theoretical calculations to investigate such difficult crystal structures.

## Supporting Information Available

X-ray data collection details, histogram and frequency table for Au···Au interactions, ADPs, bond lengths and valence angles, Hirshfeld surface and NCI analysis, relative contributions of different intermolecular contacts to the Hirshfeld surface, 2D fingerprint plots for the Hirshfeld surface, NCI isosurfaces (*s* = 0.3 au) for the investigated dimer, HAR refinement details, effects of a different basis set used in HAR, QTAIM parameters at the BCPs at different levels of theory, labeling scheme and molecular graphs for the investigated dimer, 1D plots of the electron density as a function of the length of the Au···Au contact, normal probability plots for tested models, anharmonic thermal motions analysis, and references (PDF)

## Accession Codes

CCDC 2116322–2116325 contain the supplementary crystallographic data for this paper. These data can be obtained free of charge via www.ccdc.cam.ac.uk/data_request/cif, or by emailing data_request@ccdc.cam.ac.uk, or by contacting The Cambridge Crystallographic Data Centre, 12 Union Road, Cambridge CB2 1EZ, UK; fax: +44 1223 336033.
