# Supplementary material for: Aurophilic Interactions Studied by Quantum Crystallography
Source: Inorg Chem. 2022 Mar 1;61(10):4235–9. doi: 10.1021/acs.inorgchem.1c03333 (PMC8924918; doi:10.1021/acs.inorgchem.1c03333)
Supplement: Supplementary file 1 — ic1c03333_si_001.pdf [file ic1c03333_si_001.pdf]

Aurophilic Interactions Studied by Quantum Crystallography

Sylwia Pawlędzio[a], Maura Malinska[a], Florian Kleemiss[c], Simon Grabowsky[b], Krzysztof Woźniak[a*]

[a] Biological and Chemical Research Centre, Department of Chemistry, University of Warsaw, Żwirki i Wigury 101, 02-089 Warszawa, Poland

[b] Department of Chemistry, Biochemistry and Pharmaceutical Sciences, University of Bern, Freiestrasse 3, Bern 3012, Switzerland.

[c] Faculty for Chemistry und Pharmacy, University of Regensburg, Universitätsstr. 31, 93053 Regensburg, Germany.

*Corresponding Author: kwozniak@chem.uw.edu.pl

Experimental

The chloro(dimethyl sulfide)gold(I) was purchased from Sigma-Aldrich company (batch No. 420727). The needle-like single crystal was selected directly from the commercial reagent and used for the X-ray diffraction data collection. The measurement was performed using a Huber 1/4χ-axis diffractometer equipped with a Pilatus3 X 1M CdTe (P3) detector. Data collection was performed at 80 K. The X-ray radiation wavelength of 0.2486 Å was used as an incident beam. The Pilatus frames were converted to the Bruker .sfrm format using published software[1] and integrated using APEX3[2]. Absorption correction was performed by multi-scan method implemented in SADABS[3]. The resolution of obtained data was 0.65 Å.

Table S1. The X-ray data collection details.

Empirical formula	C2 H6 Au Cl S
Formula weight	294.555
Crystal system	monoclinic
Space group	$P\ 2_1/c$
Z	4
F(000)	520
Radiation/Å	0.2482
a/Å	5.9885(4)
b/Å	14.6568(10)
c/Å	6.2791(4)
β/°	95.9891(15)
Volume/Å^3	548.12(6)
Temperature/K	80
Absorption correction	multi-scan
ρ_{calc}g/cm^3	3.569
μ/mm^{-1}	1.781
Crystal size/mm^3	0.079x0.025x0.021
2θ range for data collection/°	1.944 to 22.048
Index ranges	$-9 \le h \le 9$<br>$-22 \le k \le 22$<br>$-9 \le l \le 9$
Reflections collected	14397
Independent reflections	2042
R_{int}	4.38%
R_{sigma}	4.60%

Cambridge Structural Database (CSD) search

In order to examine the mean values of structural parameters characterizing intermolecular aurophilic interactions, CSD[4] was searched with the QUEST program[5]. The Au···Au intermolecular contact was specified to be shorter than the sum of their van der Waals radii and the search query was performed for single crystal structures with determined 3D coordinates. As a result, 1166 structures were found. The search in CSD clearly showed a differences in the frequency distribution of the distances of aurophilic contacts, which is showed in Figure S1. The mean value is 3.16 Å (Table S2).

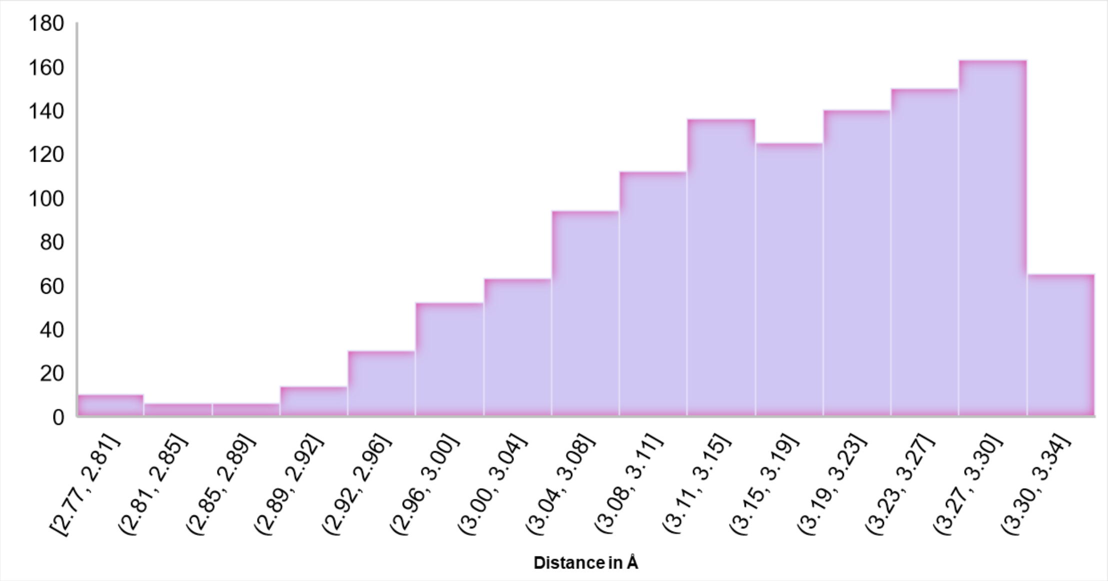

Figure S1. The histogram of CSD search results. The *y*-axis represents number of structures that fall into a specific range of Au···Au distance (*x*-axis).

Table S2. Statistics of frequency distribution given in Figure S1.

**Mean**	3.16
**Median**	3.17
**Min. value**	2.77
**Max. value**	3.32

IAM refinement details

The structure was solved by direct methods[6] and refined by the weighted full matrix least-squares on F^2 technique using *SHELXL*[7] within the graphical interface of *Olex2*[8]. The H atoms were positioned geometrically, with C—H = 0.98 A°, and constrained to ride on their parent atoms, with $U_{iso}(H) = 1.5U_{eq}(C)$.

Table S3. Anisotropic Displacement Parameters (Å^2×10^3) after IAM.

Atom	U11	U22	U33	U23	U13	U12
**Au1**	11.21(9)	8.38(9)	11.86(9)	-0.75(4)	3.14(6)	-0.42(4)
**S1**	12.3(4)	11.1(4)	12.7(4)	0.2(3)	1.5(3)	0.5(3)
**Cl1**	15.4(4)	11.9(4)	17.9(4)	0.3(3)	3.7(3)	2.8(3)
**C1**	16.3(17)	10.3(16)	19.4(17)	-0.6(13)	0.9(14)	-0.6(13)
**C2**	11.8(16)	17.2(19)	22.7(19)	-0.2(15)	5.4(15)	0.6(13)

Table S4. Interatomic Distances and Bond Lengths after IAM.

Atom	Atom	Length/Å	Atom	Atom	Length/Å
**Au1**	**Au1[1]**	3.15891(19)	**Au1**	**Cl1**	2.2858(10)
**Au1**	**Au1[2]**	3.15893(19)	**S1**	**C1**	1.811(5)
**Au1**	**S1**	2.2685(11)	**S1**	**C2**	1.801(5)

[1]+X,3/2-Y,-1/2+Z;[2]+X,3/2-Y,1/2+Z

Table S5. Interatomic Distances and Bond Angles after IAM.

Atom	Atom	Atom	Angle/°	Atom	Atom	Atom	Angle/°
**Au1[1]**	**Au1**	**Au1[2]**	167.305(11)	**Cl1**	**Au1**	**Au1[2]**	90.14(3)
**S1**	**Au1**	**Au1[2]**	93.16(3)	**C1**	**S1**	**Au1**	105.09(15)
**S1**	**Au1**	**Au1[1]**	76.84(3)	**C2**	**S1**	**Au1**	107.97(16)
**S1**	**Au1**	**Cl1**	176.66(4)	**C2**	**S1**	**C1**	100.4(2)
**Cl1**	**Au1**	**Au1[1]**	99.96(3)				

[1]+X,3/2-Y,-1/2+Z; [2]+X,3/2-Y,1/2+Z

Hirshfeld surface and NCI analysis

Hirshfeld surface[9] is a method in which space around molecules in the crystal is obtained by partitioning electron density into non-overlapping molecular fragments. The isosurface encloses region, where the ratio of promolecule to procrystal electron densities is equal to 0.5. Hirshfeld surface is mapped by the normalized contact distance (d_{norm}), which is defined by the closest internal (d_i) and external (d_e) distances of atoms to the surface and their van der Waals radii. The 2D-fingerprint of the Hirshfeld surface represents combination of de and d_i in form of the two-dimensional plot.

To evaluate qualitatively and quantitively the intermolecular interactions present in the crystal, we used molecular Hirshfeld surface analysis and 2D fingerprint plots[9,10] (Figs. S1 and S3) associated with it. The fingerprint plot decomposition into contributions of particular intermolecular contacts to the Hirshfeld surface area confirmed the presence of Au···Au, Au···H, Au···Cl, Au···S interactions as well as short H···Cl, H···H, H···S and S···Cl contacts in the supramolecular architecture (Fig. S2). The aurophilic interaction in the Au···Au dimer was accompanied by C–H···Cl, C–H···Au and C–S···Au short interatomic contacts (Figs. S1–3). Using NCI analysis[11] we confirmed the attractive character of the Au···Au interaction, which is visible as bluish tones on the NCI isosurface. All other intermolecular contacts present in the Au···Au dimer have a weak dispersive character, which is exhibited as a green, solid isosurface (Fig. S4) and can be estimated to have a magnitude of *ca.* -54.4 kcal/mol (Table 2).

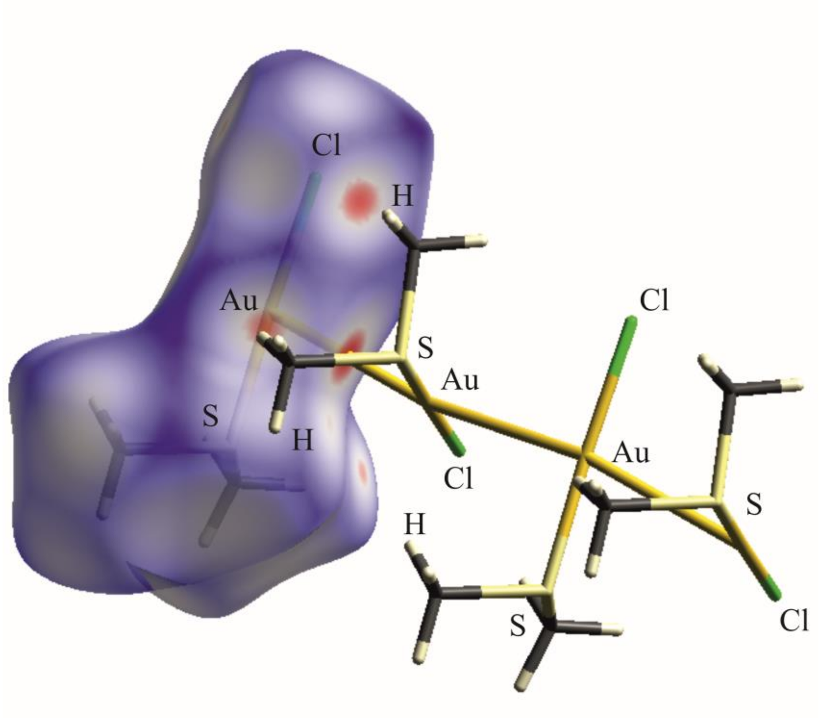

Figure S2. The Hirshfeld surface of the compound mapped with d_{norm}. The red color represents the area on the surface where intermolecular contacts between atoms are closer than the sum of their van der Waals radii. The Hirshfeld surface analysis was carried out for the geometry obtained after HAR (rks_b3lyp_anh model with x2c-TZVPPall basis set).

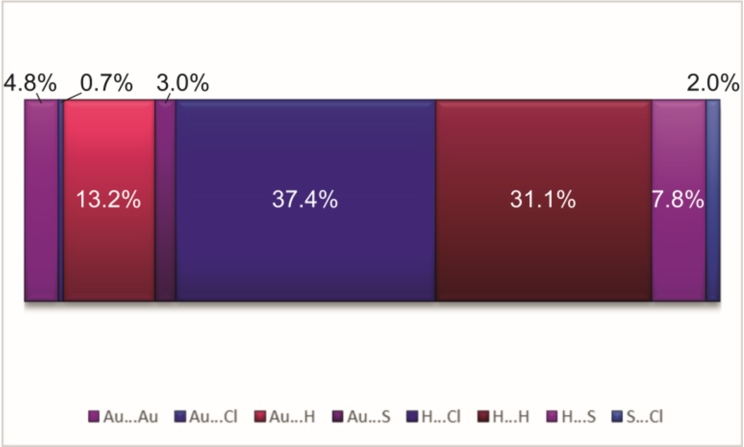

Figure S3. Relative contributions of different intermolecular contacts to the Hirshfeld surface area in the investigated compound.

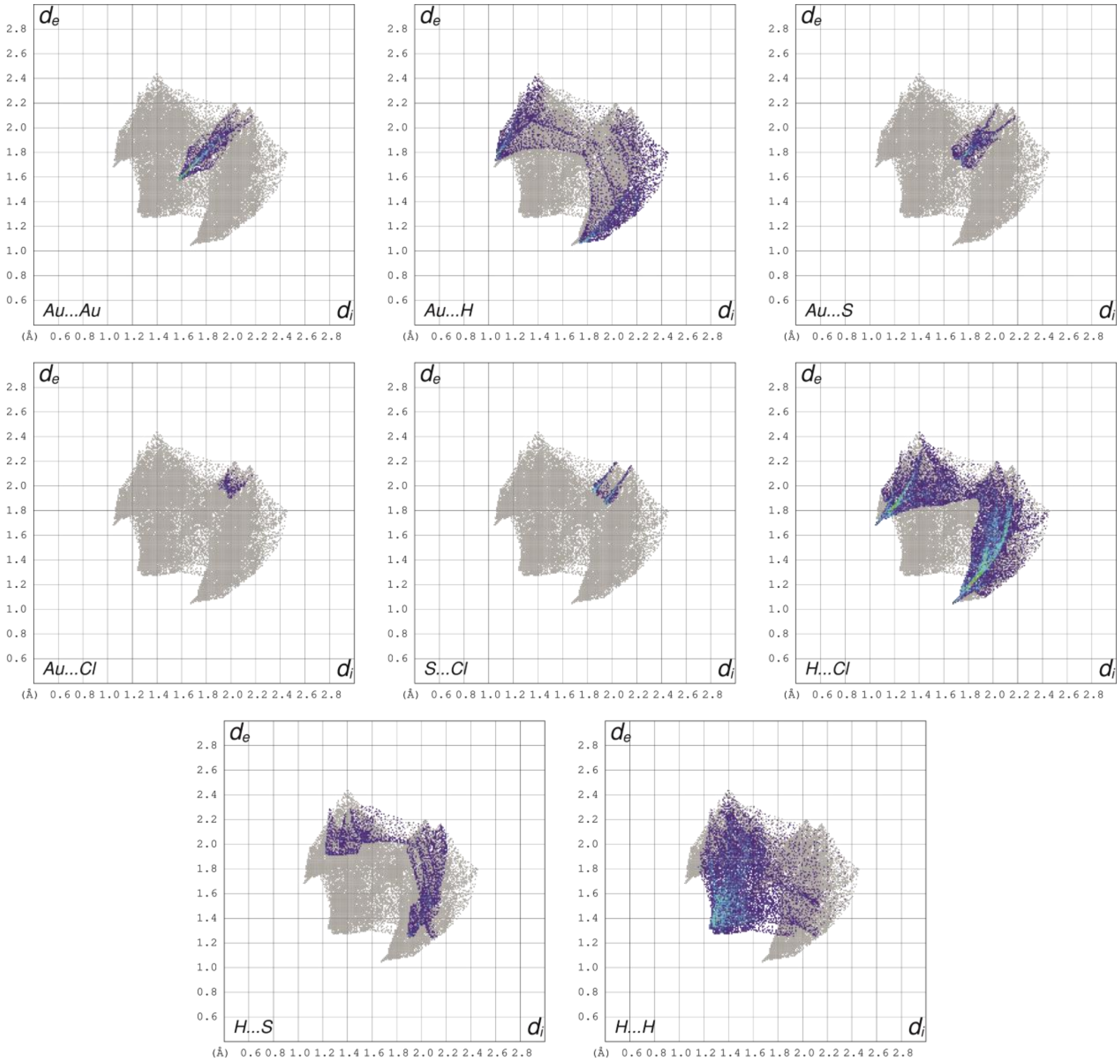

Figure S4. The 2D-fingerprint of the Hirshfeld surface showing various intermolecular contacts in the investigated compound.

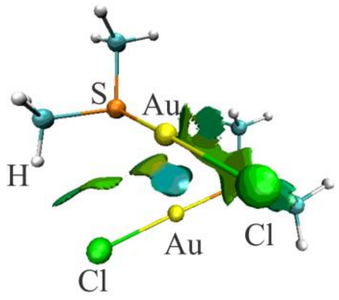

Figure S5. The NCI isosurfaces (s = 0.3 a.u.) for the investigated dimer. Green area corresponds to sign(λ_2)ρ values near zero, which indicates weak attractive van der Waals interactions. Blue area corresponds to the negative sign(λ_2)ρ, indicating more attractive forces.

HAR refinements details

The geometry obtained from IAM was further used in HAR[12]. Similarly to our previous study[13], the impact of the electron correlation and relativistic effects was investigated based on the molecular wavefunction calculated using the HF or B3LYP-DFT methods. The segmented contracted all-electron basis sets of triple-ζ valence quality optimized at the x2c level (x2c-TZVPPall[14]) was chosen. The QTAIM analysis was performed using AimAll[15]. In this study, aspherical scattering factors were obtained either at, HF or DFT, levels of theory. Calculations of the molecular wavefunction were carried out for the dimer of molecules. To investigate effect of the electron correlation and relativity B3LYP functional and second-order DKH (DKH2) Hamiltonian were used, respectively. The thermal motions of atoms were refined anisotropically for C, S and Cl. For the Au atom, an anharmonic thermal motions were refined up to fourth-order Gram–Charlier (GC) coefficients[16]. Thermal motion of H atoms was treated isotropically. During HAR, atomic positions of Au, C, S and Cl were refined without any restrains and constrains. All H atoms were positioned geometrically and refined using a riding model by HFIX 137, where the torsion angles were allowed to refine, while X-H distances were elongated to the neutron values and fixed, together with the Y-X-H angles. The exception was rks_anh_nrel model in which, keeping C–H distances to the corresponding neutron values was not possible. The values of the Δf′ and Δf′′ for the anomalous dispersion correction were taken from the Sasaki table[17]. Consequently, following models were tested:

- b3lyp_rks_anh_nrel
- b3lyp_rks_anh_rel
- hf_anh_rel

where nrel denotes the non-relativistic method, and rel refer to the DKH2 approach.

Table S6. Structure refinement details. Final R indices are provided for the HAR models performed in NoSpherA2.

	**rks_anh_nrel**	**rhf_anh_rel**	**rks_anh_rel**
**Data/restraints/parameters**	2042/0/95	2042/0/95	2042/0/95
**Goodness-of-fit on F2**	0.914	0.923	0.924
**Final R indices [I>=2σ (I)]**	R_1 = 1.98%<br>wR_2 = 5.82%	R_1 = 2.01%<br>wR_2 = 5.92%	R1 =2.01%<br>*w*R2 = 5.93%
**Final R indices [all data]**	R_1 = 2.14%<br>wR_2 = 5.97%	R_1 = 2.18%<br>wR_2 = 6.08%	R1 = 2.18%<br>*w*R2 = 6.08%
**Largest diff. peak/hole / eÅ^{-3}**	1.98/-1.73	1.92/-1.67	1.91/-1.65

Table S7. Interatomic Distances and Bond Lengths after HAR.

		**Length/Å**		
**Atom**	**Atom**	**rks_anh_nrel**	**rhf_anh_rel**	**rks_anh_rel**
**Au1**	**Au1^1**	3.1592(10)	3.1593(10)	3.1593(10)
**Au1**	**Au1^2**	3.1592(10)	3.1593(10)	3.1593(10)
**Au1**	**S1**	2.2699(10)	2.2697(11)	2.2697(11)
**Au1**	**Cl1**	2.2854(10)	2.2850(10)	2.2850(10)
**S1**	**C1**	1.810(4)	1.813(4)	1.813(4)
**S1**	**C2**	1.801(4)	1.798(4)	1.798(4)

^{1}+X,3/2-Y,1/2+Z; ^{2}+X,3/2-Y,-1/2+Z

Basis set choice

In HAR ,only all-electron basis sets can be used. Among available basis sets in NoSperA2[18], the family of the x2c basis sets is recommended, when DKH2 Hamiltonian is included. For the basis set comparison, we performed refinements at b3lyp_rks_anh_rel level of theory with Jorge-TZP-DKH, x2c-SVPall, x2c-TZVPall and x2c-TZVPPall basis sets.

The differences between tested basis sets are present in Figure S5. The biggest inequality between them is visible in the core and outer core regions. Jorge-TZP-DKH basis set is the most outstanding, which is especially visible in the region between 0.4 and 0.8 Å. The local charge depletion and concentration are shifted in the direction of the metal atom with a significantly higher local maximum and minimum, when compare to x2c-TZVPPall basis set. Although, x2c-SVPall basis set is not much bigger than Jorge-TZP-DKH basis set, the reduction of the maximum/minimum of the local charge concentration/depletion seems to be quite large. The difference between x2c-TZVPall and x2c-TZVPPall basis sets is very small, mostly seen in the region around 0.6 Å. The differences are also visible when looking at electron density at BCP (Table S5). In this study, x2c-TZVPPall basis set was chosen.

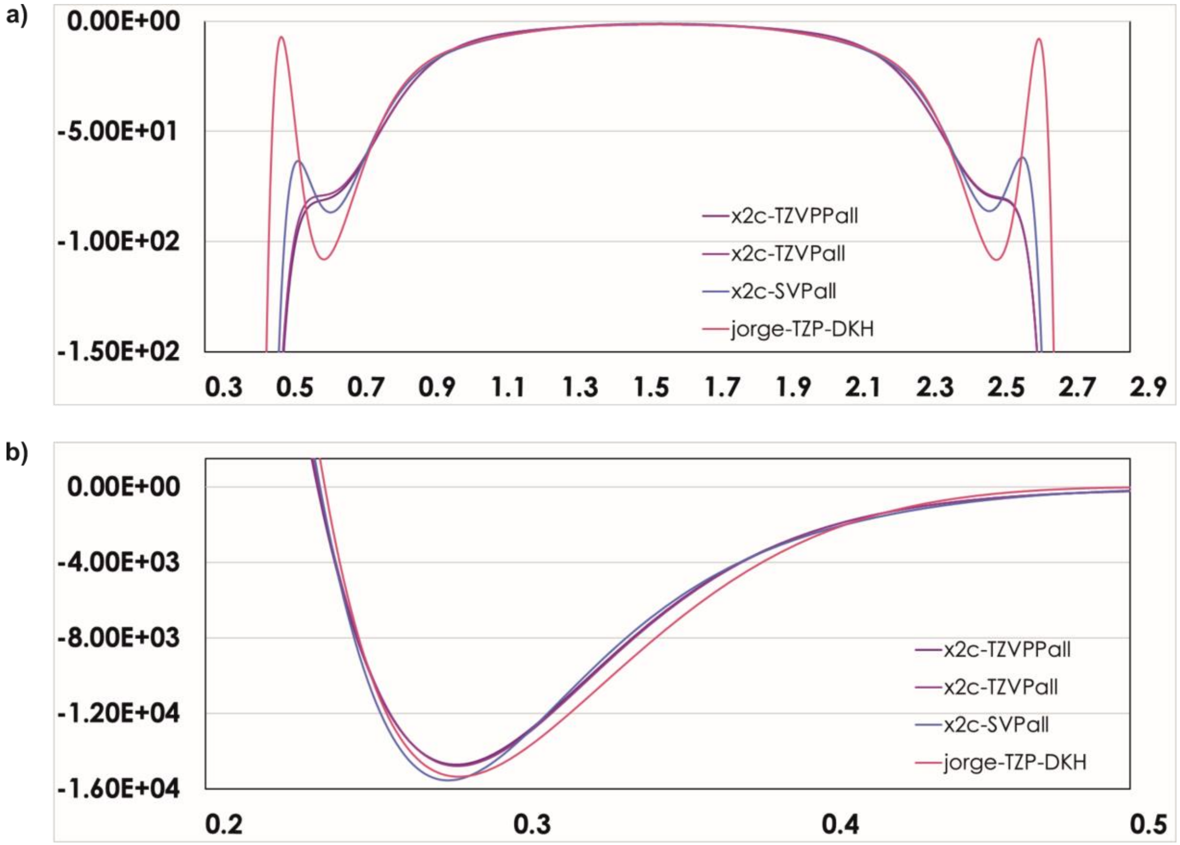

Figure S6. Effect of a different basis set used in HAR at b3lyp_rks_anh_rel level of theory on 1D plots of negative Laplacian (y-axis, in eÅ^{-5}) as a function of the the length of the Au···Au contact (x-axis, in Å): (a) 0.3 - 3Å region, (b) 0.2 – 0.5 Å region.

Table S8. QTAIM parameters at BCP for contacts present in Au···Au dimer for HAR at rks/b3lyp/rel level of theory with different basis set applied.

rks_anh_rel BCP	ρ(r) e/Å^3	∇^{2}ρ(r) e/ Å^5	ε	K hartree	G hartree	V hartree	H hartree	|V|/G	DI(A|B)
x2c-TZVPPall	0.160	1.398	0.02	0.001	0.015	-0.016	-0.001	1.046	0.25
x2c-TZVPall	0.160	1.405	0.02	0.001	0.015	-0.016	-0.001	1.039	0.25
x2c-SVPall	0.163	1.139	0.01	0.004	0.016	-0.019	-0.004	1.241	0.23
jorge-TZP-DKH	0.144	1.351	0.06	0.000	0.014	-0.015	0.000	1.018	0.21

Table S9. QTAIM parameters at BCP for contacts present in Au···Au dimer as defined in Figure 2 and delocalization index (DI) computed between two atomic basins. Symmetry codes: ^{1} x, y, z and 2 x, 1.5 – y, 0.5 +z.

Contact	Method	ρ(r) eÅ^{-3}	∇^{2}ρ(r) eÅ^{-5}	V_r hartree	H_r/ρ(r) hartree/e	|V_r|/G_r	DI
Au···H	rks_anh_rel	0.076	0.680	-0.006	0.033	0.944	0.06
	rks_anh_nrel	0.063	0.600	-0.005	0.064	0.893	0.05
	rhf_anh_rel	0.068	0.716	-0.006	0.061	0.909	0.05
^{1}Cl···^{2}H	rks_anh_rel	0.064	0.675	-0.005	0.126	0.792	0.06
	rks_anh_nrel	0.066	0.672	-0.005	0.115	0.807	0.07
	rhf_anh_rel	0.067	0.677	-0.005	0.113	0.767	0.07
^{2}Cl···^{1}H	rks_anh_rel	0.050	0.539	-0.003	0.150	0.753	0.05
	rks_anh_nrel	0.052	0.552	-0.004	0.139	0.767	0.05
	rhf_anh_rel	0.051	0.540	-0.005	0.140	0.811	0.05

Table S10. QTAIM parameters at BCP for bonds and contacts present in Au⋯Au dimer for HAR at rks/b3lyp/rel level of theory. Symmetry codes: ¹ x, y, z and 2 x, 1.5 – y, 0.5 +z. Labelling scheme is presented in Figure S7.

rks_anh_rel<br>BCP	ρ(r)<br>e/Å³	∇²ρ(r)<br>e/ Å⁵	ε	K<br>hartree	G<br>hartree	V<br>hartree	H<br>hartree	|V|/G	DI(A|B)
¹Au1⋯²Au1	0.160	1.398	0.025	0.001	0.015	-0.016	-0.001	1.046	0.250
¹Au1 – ¹S1	0.785	4.504	0.005	0.052	0.099	-0.150	-0.052	1.526	1.024
²Au1⋯¹H2b	0.076	0.680	0.059	0.000	0.007	-0.006	0.000	0.944	0.060
²Au1 – ²S1	0.785	4.539	0.004	0.052	0.099	-0.151	-0.052	1.524	0.998
¹Au1 – ¹Cl1	0.709	5.218	0.013	0.040	0.094	-0.134	-0.040	1.425	1.042
²Au1 – ²Cl1	0.711	5.140	0.017	0.040	0.094	-0.134	-0.040	1.430	1.058
¹S1 – ¹C1	1.245	-7.674	0.055	0.128	0.049	-0.177	-0.128	3.639	1.051
²Cl1⋯¹H1a	0.064	0.675	0.055	-0.001	0.006	-0.005	0.001	0.792	0.061
²S1 – ²C1	1.245	-7.681	0.048	0.128	0.048	-0.177	-0.128	3.645	1.043
¹Cl1⋯²H1b	0.050	0.539	0.075	-0.001	0.004	-0.003	0.001	0.753	0.047
¹C1 – ¹H1a	1.982	-26.593	0.012	0.316	0.040	-0.357	-0.316	8.823	0.909
²C1 – ²H1a	1.962	-25.887	0.017	0.313	0.044	-0.357	-0.313	8.088	0.950
¹C1 – ¹H1b	1.953	-25.819	0.015	0.311	0.043	-0.355	-0.311	8.171	0.953
²C1 – ²H1b	1.971	-26.489	0.011	0.315	0.040	-0.355	-0.315	8.877	0.923
¹C1 – ¹H1c	1.950	-25.583	0.016	0.311	0.045	-0.356	-0.311	7.838	0.955
²C1 – ²H1c	1.951	-25.612	0.017	0.311	0.045	-0.356	-0.311	7.863	0.954
¹S1 – ¹C2	1.281	-8.267	0.057	0.136	0.050	-0.186	-0.136	3.706	1.060
²S1 – ²C2	1.284	-8.318	0.054	0.137	0.050	-0.187	-0.137	3.712	1.056
¹C2 – ¹H2a	1.952	-25.718	0.012	0.311	0.044	-0.355	-0.311	8.038	0.952
²C2 – ²H2a	1.952	-25.752	0.009	0.311	0.044	-0.355	-0.311	8.071	0.952
¹C2 – ¹H2b	1.966	-26.024	0.011	0.313	0.043	-0.357	-0.313	8.215	0.918
²C2 – ²H2b	1.962	-25.884	0.010	0.313	0.044	-0.357	-0.313	8.094	0.948
¹C2 – ¹H2c	1.953	-25.682	0.013	0.311	0.045	-0.356	-0.311	7.929	0.952
²C2 – ²H2c	1.955	-25.709	0.011	0.312	0.045	-0.357	-0.312	7.939	0.952

Table S11. QTAIM parameters at BCP for bonds and contacts present in Au⋯Au dimer for HAR at rks/b3lyp/nrel level of theory. Symmetry codes: ¹ x, y, z and 2 x, 1.5 – y, 0.5 +z. Labelling scheme is presented in Figure S7.

rks_anh_nrel<br>BCP	ρ(r)<br>e/Å³	∇²ρ(r)<br>e/ Å⁵	ε	K<br>hartree	G<br>hartree	V<br>hartree	H<br>hartree	|V|/G	DI(A|B)
¹Au1⋯²Au1	0.125	1.133	0.063	0.000	0.011	-0.011	0.000	0.975	0.165
¹Au1 – ¹S1	0.667	7.810	0.013	0.032	0.113	-0.145	-0.032	1.284	0.870
²Au1⋯¹H2b	0.734	7.089	0.028	0.042	0.116	-0.158	-0.042	1.364	0.832
²Au1 – ²S1	0.667	7.777	0.019	0.032	0.113	-0.145	-0.032	1.286	0.888
¹Au1 – ¹Cl1	0.734	6.984	0.025	0.043	0.115	-0.158	-0.043	1.370	0.812
²Au1 – ²Cl1	1.952	-25.454	0.019	0.309	0.045	-0.354	-0.309	7.895	0.954
¹S1 – ¹C1	1.972	-26.591	0.010	0.316	0.040	-0.356	-0.316	8.910	0.922
²Cl1⋯¹H1a	1.946	-25.356	0.022	0.308	0.045	-0.353	-0.308	7.879	0.953
²S1 – ²C1	1.948	-25.619	0.010	0.310	0.044	-0.355	-0.310	7.986	0.954
¹Cl1⋯²H1b	1.957	-25.678	0.011	0.311	0.045	-0.356	-0.311	7.948	0.951
¹C1 – ¹H1a	1.953	-25.562	0.015	0.310	0.045	-0.354	-0.310	7.956	0.951
²C1 – ²H1a	1.982	-26.548	0.014	0.316	0.041	-0.357	-0.316	8.719	0.912
¹C1 – ¹H1b	1.947	-25.642	0.014	0.310	0.044	-0.353	-0.310	8.086	0.955
²C1 – ²H1b	1.946	-25.407	0.019	0.309	0.045	-0.353	-0.309	7.864	0.954
¹C1 – ¹H1c	1.950	-25.408	0.010	0.308	0.045	-0.353	-0.308	7.873	0.928
²C1 – ²H1c	1.951	-25.513	0.015	0.309	0.044	-0.353	-0.309	7.988	0.951

**^{1}S1 – ^{1}C2**	1.951	-25.688	0.010	0.311	0.044	-0.355	-0.311	8.014	0.952
**^{2}S1 – ^{2}C2**	0.051	0.540	0.111	-0.001	0.005	-0.003	0.001	0.767	0.050
**^{1}C2 – ^{1}H2a**	0.063	0.600	0.021	-0.001	0.006	-0.005	0.001	0.893	0.047
**^{2}C2 – ^{2}H2a**	0.067	0.677	0.050	-0.001	0.006	-0.005	0.001	0.811	0.067
**^{1}C2 – ^{1}H2b**	1.237	-7.549	0.078	0.127	0.049	-0.176	-0.127	3.602	1.061
**^{2}C2 – ^{2}H2b**	1.274	-8.152	0.083	0.136	0.051	-0.187	-0.136	3.649	1.075
**^{1}C2 – ^{1}H2c**	1.237	-7.531	0.079	0.127	0.049	-0.176	-0.127	3.591	1.072
**^{2}C2 – ^{2}H2c**	1.272	-8.145	0.086	0.136	0.051	-0.187	-0.136	3.656	1.081

Table S12. QTAIM parameters at BCP for bonds and contacts present in Au⋯Au dimer for HAR at rhf/b3lyp/rel level of theory. Symmetry codes: ^{1} x, y, z and 2 x, 1.5 – y, 0.5 +z. Labelling scheme is presented in Figure S7.

**hf_anh_rel BCP**	**$\rho(r)$ e/Å^3**	**$\nabla^2\rho(r)$ e/ Å^5**	**ε**	**K hartree**	**G hartree**	**V hartree**	**H hartree**	**|V|/G**	**DI(A|B)**
**^{1}Au1⋯^{2}Au1**	0.140	1.502	0.026	0.000	0.015	-0.015	0.000	0.986	0.184
**^{1}Au1 – ^{1}S1**	0.705	5.863	0.013	0.042	0.102	-0.144	-0.042	1.406	0.925
**^{2}Au1⋯^{1}H2b**	0.794	5.163	0.009	0.055	0.108	-0.163	-0.055	1.504	0.944
**^{2}Au1 – ^{2}S1**	0.708	5.768	0.015	0.042	0.102	-0.144	-0.042	1.412	0.941
**^{1}Au1 – ^{1}Cl1**	0.068	0.716	0.049	-0.001	0.007	-0.006	0.001	0.909	0.048
**^{2}Au1 – ^{2}Cl1**	0.794	5.196	0.009	0.055	0.109	-0.163	-0.055	1.504	0.922
**^{1}S1 – ^{1}C1**	1.997	-28.228	0.013	0.334	0.042	-0.376	-0.334	9.050	0.959
**^{2}Cl1⋯^{1}H1a**	2.013	-28.541	0.013	0.337	0.041	-0.378	-0.337	9.208	0.934
**^{2}S1 – ^{2}C1**	2.002	-28.300	0.014	0.336	0.042	-0.378	-0.336	8.996	0.959
**^{1}Cl1⋯^{2}H1b**	1.997	-28.240	0.011	0.334	0.041	-0.376	-0.334	9.065	0.960
**^{1}C1 – ^{1}H1a**	2.008	-28.439	0.013	0.337	0.041	-0.378	-0.337	9.112	0.956
**^{2}C1 – ^{2}H1a**	2.002	-28.314	0.014	0.336	0.042	-0.378	-0.336	8.993	0.959
**^{1}C1 – ^{1}H1b**	1.998	-28.317	0.017	0.334	0.041	-0.375	-0.334	9.214	0.960
**^{2}C1 – ^{2}H1b**	1.996	-28.166	0.019	0.335	0.043	-0.378	-0.335	8.826	0.962
**^{1}C1 – ^{1}H1c**	2.033	-29.263	0.014	0.340	0.037	-0.377	-0.340	10.235	0.920
**^{2}C1 – ^{2}H1c**	2.007	-28.428	0.020	0.337	0.042	-0.378	-0.337	9.076	0.958
**^{1}S1 – ^{1}C2**	2.021	-29.119	0.013	0.338	0.036	-0.375	-0.338	10.324	0.930
**^{2}S1 – ^{2}C2**	1.997	-28.197	0.020	0.335	0.043	-0.378	-0.335	8.860	0.961
**^{1}C2 – ^{1}H2a**	0.048	0.582	0.077	-0.001	0.005	-0.004	0.001	0.747	0.040
**^{2}C2 – ^{2}H2a**	0.061	0.729	0.047	-0.001	0.006	-0.005	0.001	0.783	0.053
**^{1}C2 – ^{1}H2b**	1.347	-10.478	0.061	0.154	0.045	-0.199	-0.154	4.395	1.054
**^{2}C2 – ^{2}H2b**	1.309	-9.802	0.056	0.146	0.044	-0.190	-0.146	4.302	1.041
**^{1}C2 – ^{1}H2c**	1.351	-10.548	0.059	0.155	0.045	-0.200	-0.155	4.412	1.052
**^{2}C2 – ^{2}H2c**	1.309	-9.801	0.048	0.146	0.044	-0.190	-0.146	4.310	1.033

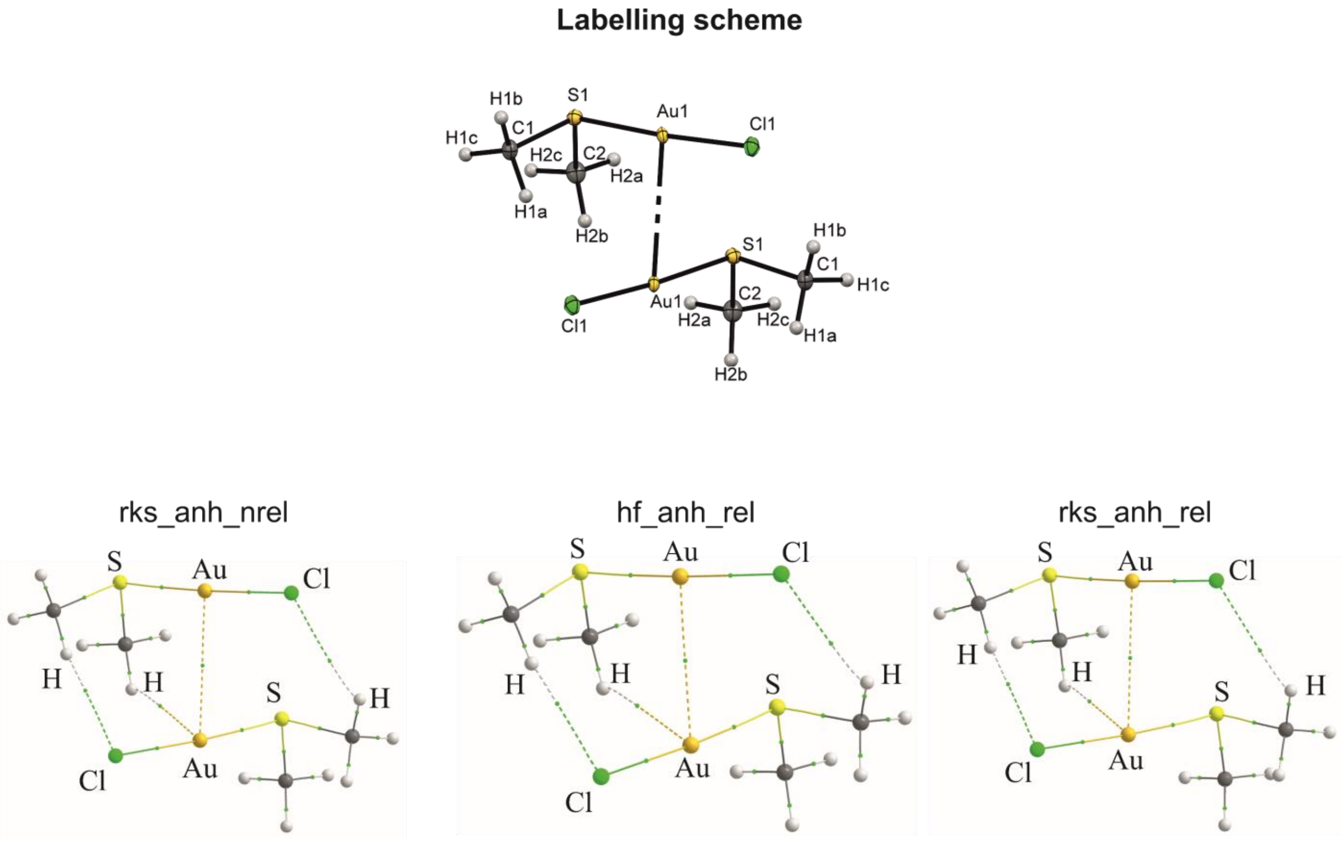

Figure S7. Labelling scheme and molecular graphs for the investigated dimer obtained from QTAIM analysis for HAR models. Lines correspond to bond paths between covalently bonded atoms, dashed lines correspond to the bond paths for non-covalent contacts, and green dots represent bond critical points.

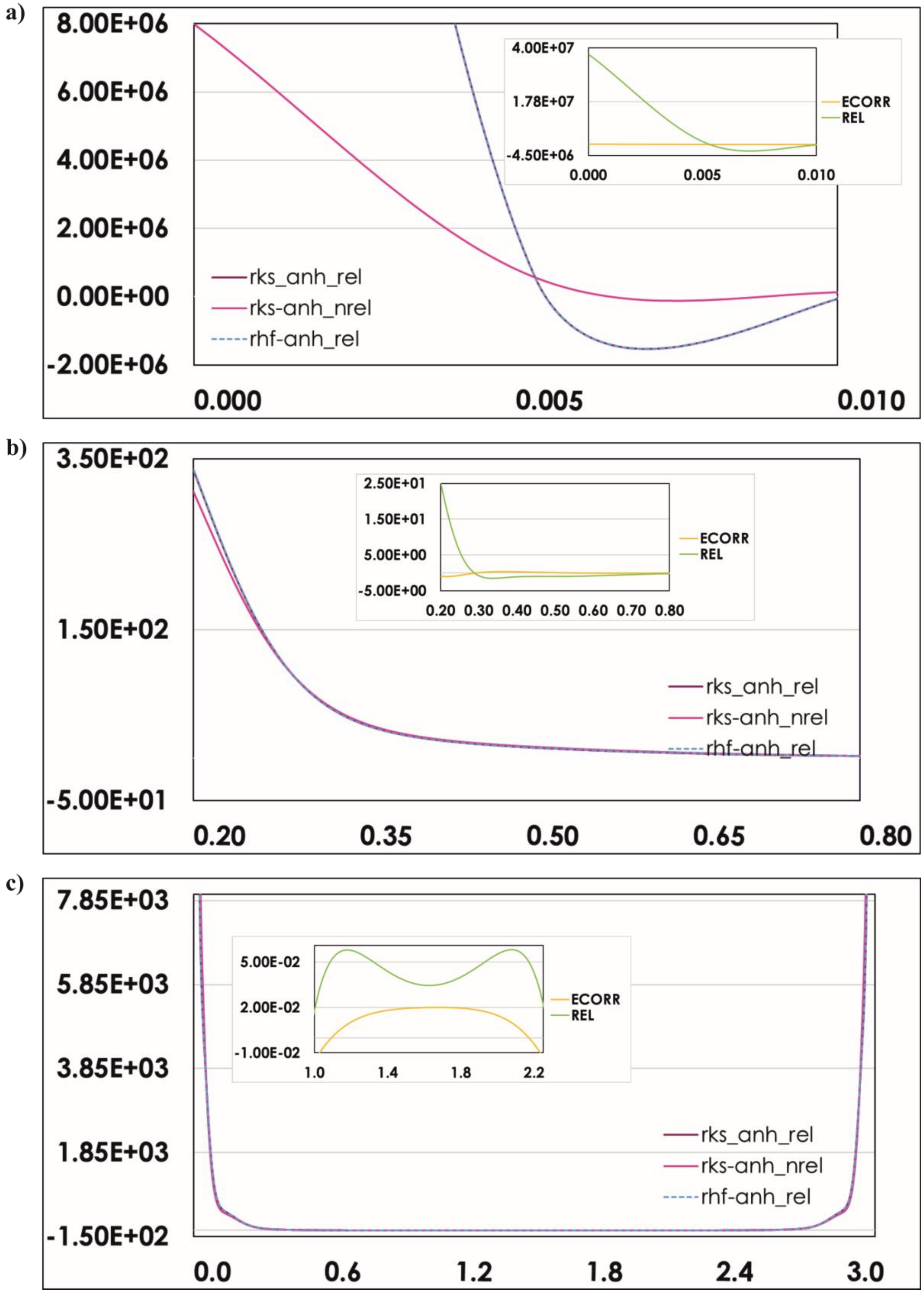

Figure S8. 1D plots of electron density (Y-axis, in eÅ^{-3}) as a function of the length of the Au···Au contact (X-axis, in Å) for the obtained HAR models, showing the most interesting changes in its course.. The subplots show difference electron densities resulting from the relativistic effects (REL) and electron correlation (ECORR), obtained as a result of the subtraction of the rel_anh_rel – rks_anh_nrel and rks_anh_rel – rhf_anh_rel models, respectively: (a) 0.0 – 0.01Å region, (b) 0.2 – 0.8 Å region, (c) 0.0 - 3.0Å region.

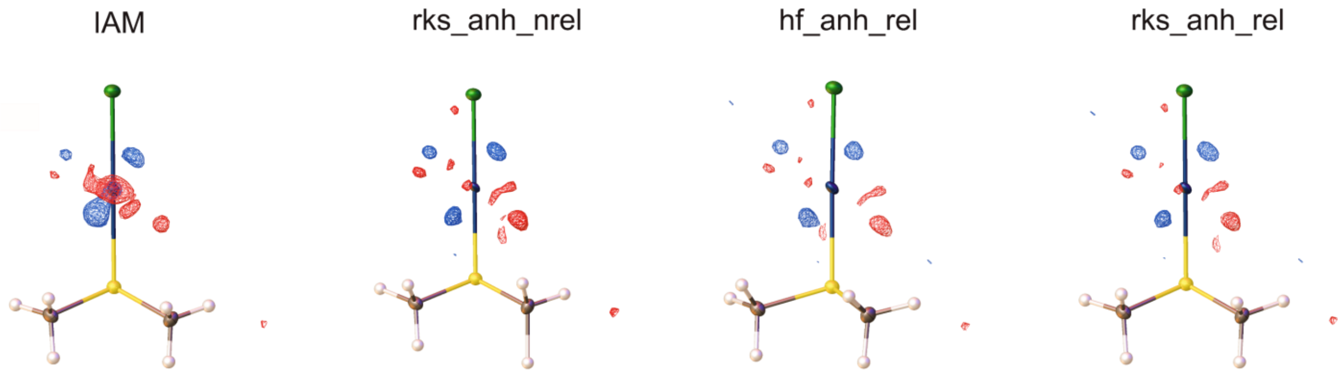

Figure S9. Residual density maps for the tested models. Contour level: 0.1eÅ^{-3}. Colours: red –positive, blue –negative.

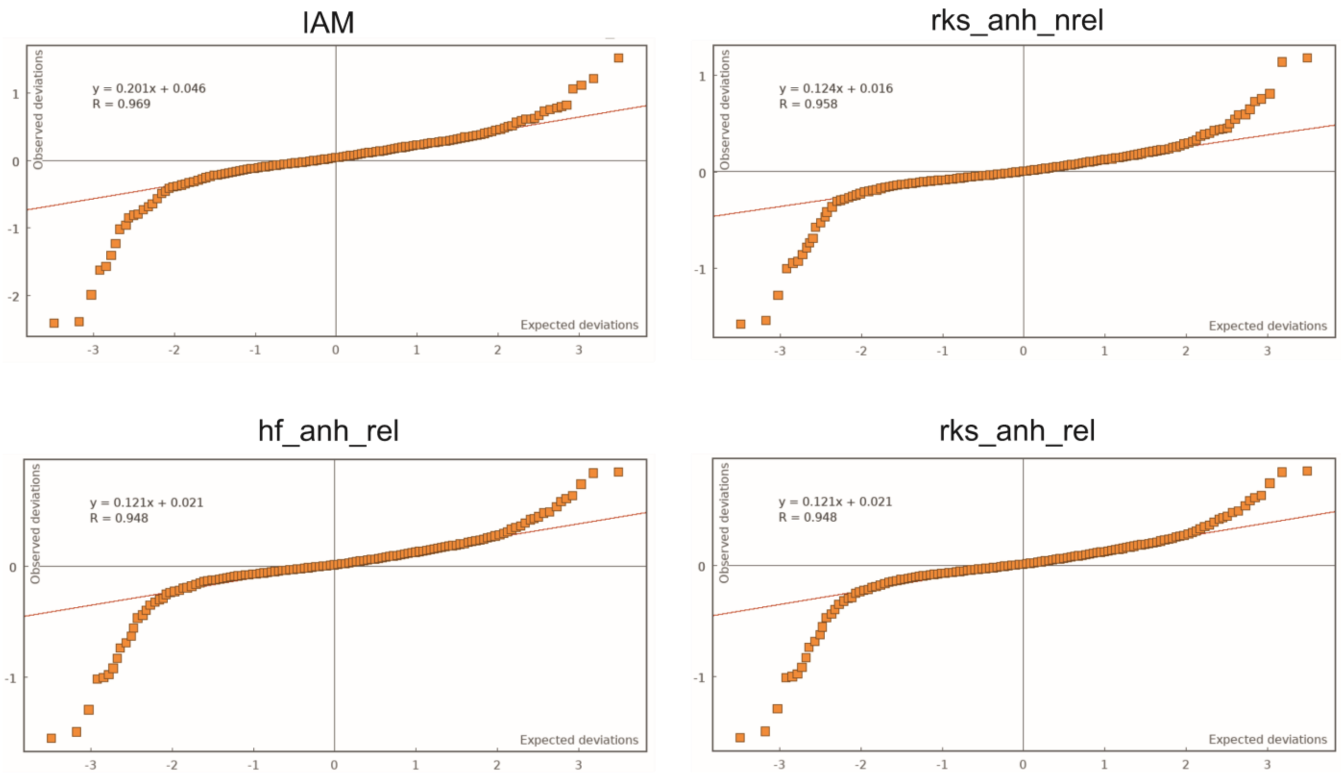

Figure S10. Normal probability plots for the tested models.

Anharmonic thermal motions analysis:

In this study we applied anharmonic approximation to describe the atomic probability density function by using Gram-Charlier (GC) expansion[16]. The use of GC expansion requires special attention. In order to prove how important is to refine anharmonic vibrations, certain criteria must be met:

1. noticeable changes in the maximum positive and negative residual densities after applying GC expansion;
2. satisfied Kuhs' rule at least for all light elements[19];
3. the probability density functions (p.d.f) for refinement with anharmonic nucleus motion should show only positive probabilities with no visible negative area;
4. at least one of the G-C coefficients should be higher than the 3σ for the refined atom.

The maximum positive and negative residual densities for the refinement with harmonic nuclear motions is significantly higher than for the HAR refinement with anharmonic approximation (Table 1). Although, the minimum data resolution required for Au was not achieved (Table S13), we found some of the G-C coefficients to be larger than three standard uncertainties (Tables S14-15). Derived the total p.d.f.s for refinements with anharmonic nuclear motion of Au up to the fourth order showed only positive integrated probability and no visible negative region around Au in the graphical representation (Fig. S11). According to the paper published by Herbs-Irmer *et al.*[20], where authors stated that *"Kuhs' rule for minimum resolutions seems to be too strict, at least for heavier elements such as sulfur and phosphorus."*, we indicated the presence of anharmonic vibrations and confirmed their physical relevance despite the too low resolution of the data.

Table S13. The minimum data resolution required (in Å^{-1}), according to the Kuhs equation (ref) and numbers of values of Gram–Charlier coefficients larger than 3 σ for anharmonic refinements.

	res.	par.
**exp.**	0.77	
**3rd**	1.31	2
**4th**	1.51	5

Table S14. 3rd order of the Gram-Charlier coefficients. Values higher than three standard uncertainties are highlighted in orange.

	rks-anh_nr	rhf-anh_rel	rks-anh_rel
U_{111}	-0.0000006(2)	-0.0000006(2)	-0.0000006(2)
U_{222}	0.00000012(4)	0.00000013(4)	0.00000013(4)
U_{333}	0.00000003(10)	0.00000006(11)	0.00000006(11)
U_{112}	-0.000000011(14)	-0.000000012(15)	-0.000000012(15)
U_{113}	0.00000005(2)	0.00000005(2)	0.00000005(2)
U_{122}	0.00000011(9)	0.00000014(10)	0.00000014(10)
U_{223}	-0.000000100(12)	-0.000000105(13)	-0.000000105(13)
U_{133}	0.000000018(13)	0.000000023(14)	0.000000023(14)
U_{233}	0.00000010(4)	0.00000010(4)	0.00000010(4)
U_{123}	0.00000085(18)	0.0000009(2)	0.0000009(2)

Table S15. 4th order of the Gram-Charlier coefficients. Values higher than three standard uncertainties are highlighted in orange.

	rks-anh_nr	rhf-anh_rel	rks-anh_rel
U_{1111}	0.00000009(4)	0.00000023(4)	0.00000023(4)
U_{2222}	-0.000000002(5)	-0.000000005(5)	-0.000000005(5)
U_{3333}	0.000000044(16)	0.000000063(18)	0.000000063(18)
U_{1112}	-0.0000000071(17)	0.0000000000(19)	0.0000000000(19)
U_{1113}	0.000000001(2)	0.000000000(2)	-0.000000000(2)
U_{1222}	-0.000000012(11)	0.000000031(13)	0.000000032(13)
U_{2223}	-0.0000000006(7)	-0.0000000011(7)	-0.0000000011(7)
U_{1333}	-0.0000000003(10)	0.0000000007(11)	0.0000000006(11)
U_{2333}	0.000000003(2)	0.000000003(2)	0.000000003(2)
U_{1122}	0.000000033(14)	0.000000049(16)	0.000000049(16)
U_{1133}	-0.0000000122(8)	-0.0000000095(9)	-0.0000000095(9)
U_{2233}	0.0000000005(6)	0.0000000003(7)	0.0000000004(7)
U_{1123}	-0.0000000099(15)	-0.0000000042(17)	-0.0000000041(17)
U_{1223}	0.000000001(4)	0.000000000(4)	0.000000000(4)
U_{1233}	0.00000016(4)	0.00000029(4)	0.00000029(4)

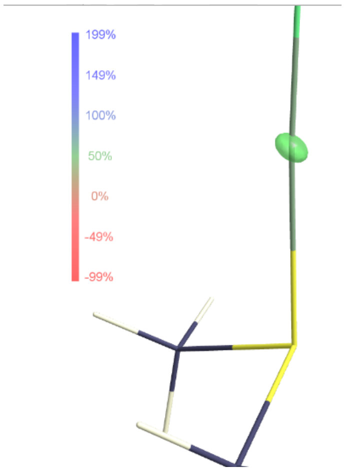

Figure S11. Graphical representation of the probability density function of 3rd and 4th order of the Gram–Charlier coefficients of the gold atom on the example of rks_anh_rel model at the 50 % probability level for all considered anharmonic refinements showing only positive regions.

References:

(1) Krause, L.; Tolborg, K.; Grønbech, T. B. E.; Sugimoto, K.; Iversen, B. B.; Overgaard, J. Accurate High-Resolution Single-Crystal Diffraction Data from a Pilatus3 X CdTe Detector. *J Appl Cryst* **2020**, *53* (3), 635–649.

(2) Bruker AXS Inc., Madison, WI. APEX3. 2016.

(3) Sheldrick, G. M. SADABS, Program for Area Detector Adsorption Correction. *University of Gottingen: Gottingen, Germany* **1996**.

(4) Groom, C. R.; Bruno, I. J.; Lightfoot, M. P.; Ward, S. C. The Cambridge Structural Database. *Acta Crystallographica B* **2016**, *72* (2), 171–179.

(5) Bruno, I. J.; Cole, J. C.; Edgington, P. R.; Kessler, M.; Macrae, C. F.; McCabe, P.; Pearson, J.; Taylor, R. New Software for Searching the Cambridge Structural Database and Visualizing Crystal Structures. *Acta Cryst B* **2002**, *58* (3), 389–397.

(6) Sheldrick, G. M. Phase Annealing in SHELX-90: Direct Methods for Larger Structures. *Acta Crystallographica Section A: Foundations of Crystallography* **1990**, *46* (6), 467–473.

(7) Sheldrick, G. M. Crystal Structure Refinement with SHELXL. *Acta Cryst C* **2015**, *71* (1), 3–8.

(8) Dolomanov, O. V.; Bourhis, L. J.; Gildea, R. J.; Howard, J. a. K.; Puschmann, H. OLEX2: A Complete Structure Solution, Refinement and Analysis Program. *Journal of Applied Crystallography* **2009**, *42* (2), 339–341.

(9) Spackman, M. A.; Jayatilaka, D. Hirshfeld Surface Analysis. *CrystEngComm* **2009**, *11* (1), 19–32.

(10) Spackman, P. R.; Turner, M. J.; McKinnon, J. J.; Wolff, S. K.; Grimwood, D. J.; Jayatilaka, D.; Spackman, M. A. CrystalExplorer: A Program for Hirshfeld Surface Analysis, Visualization and Quantitative Analysis of Molecular Crystals. *J Appl Cryst* **2021**, *54* (3), 1006–1011.

(11) Contreras-García, J.; Johnson, E. R.; Keinan, S.; Chaudret, R.; Piquemal, J.-P.; Beratan, D. N.; Yang, W. NCIPLOT: A Program for Plotting Non-Covalent Interaction Regions. *J Chem Theory Comput* **2011**, *7* (3), 625–632.

(12) Capelli, S. C.; Bürgi, H.-B.; Dittrich, B.; Grabowsky, S.; Jayatilaka, D. Hirshfeld Atom Refinement. *IUCrJ* **2014**, *1* (Pt 5), 361–379.

(13) Pawlędzio, S.; Malinska, M.; Woińska, M.; Wojciechowski, J.; Andrade Malaspina, L.; Kleemiss, F.; Grabowsky, S.; Woźniak, K. Relativistic Hirshfeld Atom Refinement of an Organo-Gold(I) Compound. *IUCrJ* **2021**, *8* (4), 608–620.

(14) Pollak, P.; Weigend, F. Segmented Contracted Error-Consistent Basis Sets of Double- and Triple-ζ Valence Quality for One- and Two-Component Relativistic All-Electron Calculations. *J. Chem. Theory Comput.* **2017**, *13* (8), 3696–3705.

(15) Keith, T. A. AIMAll (Version 19.10.12). TK Gristmill Software, Overland Park KS, USA (aim.tkgristmill.com) 2019.

(16) Mallinson, P. R.; Koritsanszky, T.; Elkaim, E.; Li, N.; Coppens, P. The Gram–Charlier and Multipole Expansions in Accurate X-Ray Diffraction Studies: Can They Be Distinguished? *Acta Crystallographica Section A* **1988**, *44* (3), 336–343.

(17) Sasaki, T.; Kasai, H.; Nishibori, E. Tightly Binding Valence Electron in Aluminum Observed through X-Ray Charge Density Study. *Scientific Reports* **2018**, *8* (1), 11964.

(18) Kleemiss, F.; Dolomanov, O. V.; Bodensteiner, M.; Peyerimhoff, N.; Midgley, L.; Bourhis, L. J.; Genoni, A.; Malaspina, L. A.; Jayatilaka, D.; Spencer, J. L.; White, F.; Grundkötter-Stock, B.; Steinhauer, S.; Lentz, D.; Puschmann, H.; Grabowsky, S. Accurate Crystal Structures and Chemical Properties from NoSpherA2. *Chem. Sci.* **2021**, *12* (5), 1675–1692.

(19) Kuhs, W. F. Generalized Atomic Displacements in Crystallographic Structure Analysis. *Acta Cryst A* **1992**, *48* (2), 80–98.

(20) Herbst-Irmer, R.; Henn, J.; Holstein, J. J.; Hübschle, C. B.; Dittrich, B.; Stern, D.; Kratzert, D.; Stalke, D. Anharmonic Motion in Experimental Charge Density Investigations. *The Journal of Physical Chemistry A* **2013**, *117* (3), 633–641.
